# Vesicouterine Fistula (Youssef Syndrome): Case Report and Literature Review

**DOI:** 10.1055/s-0038-1666998

**Published:** 2018-09

**Authors:** Reynaldo Augusto Machado Junior, Luís Carlos Machado Junior, Lúcio Lourenço e Lourenço

**Affiliations:** 1 Department of Obstetrics and Gynecology, Hospital Municipal Universitário de São Bernardo do Campo, São Bernardo do Campo, SP, Brazil; 2 Department of Gynecology, Hospital Paulistano, São Paulo, SP, Brazil

**Keywords:** cesarean section, repeat cesarean section, urinary incontinence, hematuria, cesárea, cesárea repetida, incontinência urinária, hematúria

## Abstract

**Objective** To describe a case of vesicouterine fistula and to review the literature related to this condition.

**Methods** For the review, we accessed the MEDLINE, BIREME and LILACS databases; the references of the searched articles were also reviewed.

**Results** A 38-year-old woman, in the 1st day after her 3rd cesarean, presented heavy hematuria, which was considered secondary to a difficult dissection of the bladder. A total of 6 months after delivery, she failed to resume her regular menstrual cycles and presented cyclic menouria and amenorrhea. At this time, she had two episodes of urethral obstruction by blood clots. She remained without a correct diagnosis until about two years postdelivery, when a vesicouterine fistula was confirmed through cystoscopy. A surgical correction through open abdominal route, coupled with hysterectomy, was performed. After the surgery, the symptoms disappeared. The review showed a tendency of change in the relative frequency of the different types of genitourinary fistulae. Vesicovaginal fistulae, usually caused by inadequate care during labor, are becoming less frequent than those secondary to medical procedures, such as vesicouterine fistulae. The most common cause of this latter kind of fistula is cesarean section, especially repeated cesarean sections. The diagnosis is confirmed through one or more imaging exams, or through cystoscopy. The most common treatment is surgical, and the routes are: open abdominal, laparoscopic, vaginal or robotic. There are some reports of success with the conservative treatment.

**Conclusion** Vesicouterine fistulae are becoming more common because of the increase in the performance of cesarean sections, and the condition must be considered a possible complication thereof.

## Abstract (pt)

**Resumo:**

**Objetivo** Apresentar um caso de fístula vesico-uterina e realizar revisão da literatura sobre esta condição.

**Métodos** Revisão realizada consultando-se as bases MEDLINE, BIREME e LILACS, além das referências dos artigos consultados.

**Resultados** Uma mulher de 38 anos, após sua terceira cesárea, no puerpério imediato, apresentou hematúria importante, que foi atribuída a uma dificuldade na dissecção da bexiga durante o procedimento. Seis meses pós-parto, em vez de retomar os ciclos menstruais regulares, apresentou menúria cíclica e amenorreia (síndrome de Youssef). A paciente chegou a apresentar obstrução uretral por coágulos, e permaneceu sem diagnóstico correto até cerca de anos pós-parto, quando este foi feito por cistoscopia. Ela foi então submetida a correção cirúrgica por via abdominal, associada a uma histerectomia, com desaparecimento dos sintomas. A revisão mostrou que tem havido mudança na frequência dos vários tipos de fístulas urogenitais. As fístulas vesicovaginais, normalmente secundárias à má assistência durante o parto, têm sido mais raras, enquanto aquelas secundárias a procedimentos médicos, como as vesicouterinas, têm sido mais frequentes. A causa mais comum deste tipo de fístula é a cesárea, especialmente a de repetição. A apresentação pode ser de amenorreia e menúria e/ou perda urinária. O diagnóstico é feito por um ou mais métodos de imagem ou cistoscopia. O tratamento mais comum é cirúrgico, por via abdominal aberta, laparoscópica, transvaginal ou robótica. Existem relatos de cura com tratamento conservador.

**Conclusão** As fístulas vesicouterinas têm sido mais comuns devido ao aumento da proporção de cesáreas. Deve-se ter em mente a possibilidade deste diagnóstico e considerá-las uma das possíveis complicações da cesárea.

## Introduction

Throughout time, there has been a change in the relative frequencies of genitourinary fistulae, because of a change in their causative factors. Genitourinary fistulae can be: vesicovaginal, urethrovaginal, vesicouterine, urethrocutaneous or combined (multiple) fistulae.^1^ In traditional communities, with a great frequency of out-of-hospital births, there used to be a predominance of vesicovaginal fistulae, usually secondary to a prolonged second stage of labor. With urbanization and a consequent tendency of births to occur in hospital settings, there has been a decrease in the frequency of those kinds of fistulae. They are still common, however, in rural areas with restricted access to hospital care.^2^ In regions where hospital births are the rule, there has been a relative and absolute increase in the frequency of this kind of fistulae secondary to medical interventions, such as cesarean sections, hysterectomies and others, and also secondary to malignant diseases, caused by the diseases themselves or by the interventions to treat them. The latter fistulae are more often of the urethrovaginal or vesicouterine type.^2^ Jóźwik and Jóźwik,^3^ in a series of 110 genitourinary fistulae collected from 1983 to 1994 in a urologic center in Poland, reported 50.9% of vesicovaginal, 17.3% of ureterovaginal, 13.6% of vesicouterine, and 1.8% of ureterecutaneous fistulae.^1^ Currently, it is likely that the relative frequency of vesicovaginal fistulae is even lower than the one reported in the aforementioned series, due to a continuous increase in cesarean section rates worldwide. The objective of the present study is to report a case of vesicouterine fistula and to review the literature about this specific clinical condition. The case reported is an example of Youssef Syndrome, a specific presentation of vesicouterine fistula with amenorrhea and cyclic menouria, without urinary incontinence.^4^

## Case Report

**Fig. 1:**
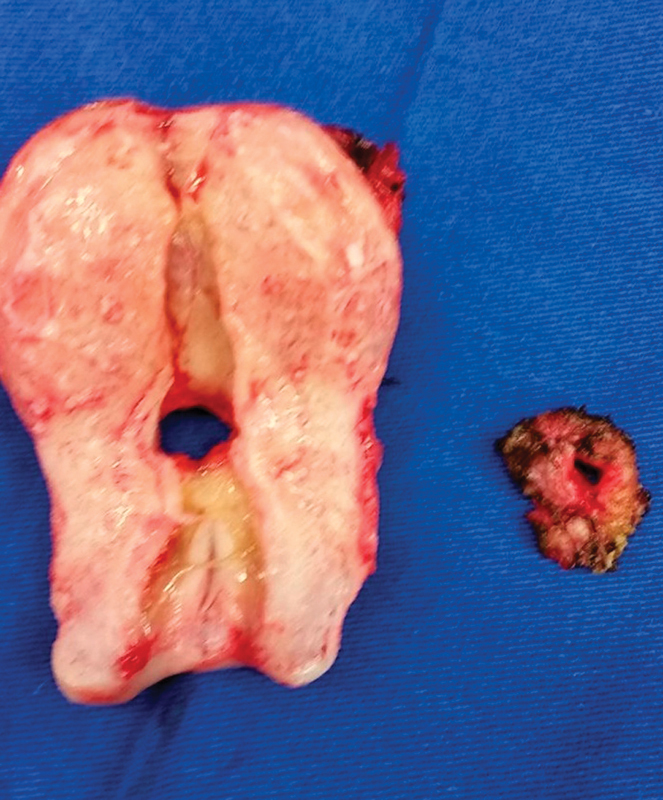
Surgical specimens showing fistulous orifices in the uterus and on the bladder wall.

A 38-year-old woman, gravida 4, with 1 spontaneous abortion and 3 cesarean births, relates that, in the 1st day after her 3rd cesarean, she had intense hematuria, requiring continuous bladder irrigation through a catheter to make the urine clear. The attending physician told her that the hematuria was a consequence of a difficult dissection of the bladder during the surgical procedure, because of the two previous cesareans. Two days after being discharged, she returned to the hospital because of a new episode of hematuria. She was then submitted to clinical and laboratory evaluations, and sent back home with the same diagnostic hypothesis: hematuria secondary to a difficult surgical dissection. She remained with persistent hematuria for three months, but did not seek further medical assistance during this period. After this period, she went through another three months without symptoms. Six months after giving birth, when she interrupted breastfeeding, instead of returning to her regular menstrual cycles, she started to have cyclic menouria, without menses, except for rare instances of scanty vaginal bleeding. This symptom persisted until one year after giving birth, and she still did not seek medical care. By this time, she had the first episode of urethral obstruction by blood clots in the urine. She went to an emergency service, and a urethral catheter was inserted, with drainage of ∼ 700 ml of heavily hematuric urine, with blood clots. About one month later, she had a similar episode, and was again treated with bladder drainage, but remained with the catheter until the end of the period that would correspond to menstruation. After these 2 episodes, she sought medical attention in an outpatient setting, where she was given oral contraception in continuous use (ethinylestradiol 20 µg plus levonorgestrel 100 µg), for unclear reasons as far as we are concerned. She remained one more year on this medication. During this period, she continued to present with some episodes of hematuria, although she did not have other episodes of urethral obstruction. She then consulted another physician, who, for the first time, raised the hypothesis of vesicouterine fistula. By this time, we could include in the differential diagnosis the following hypotheses: urinary tract lithiasis, urinary tract infection, or bladder tumor. Against lithiasis and infection, there was an absence of symptoms apart from hematuria. Benign or malignant bladder tumors could be an explanation, but the temporal relation with the last cesarean section favored fistula; the improvement of hematuria with continuous oral contraceptive also favored the latter hypothesis. Several different diagnostic procedures could have helped, but cystoscopy would be an important one. She was therefore submitted to a cystoscopy, which confirmed the diagnosis of vesicouterine fistula (Fig. 1).

**Fig. 2:**
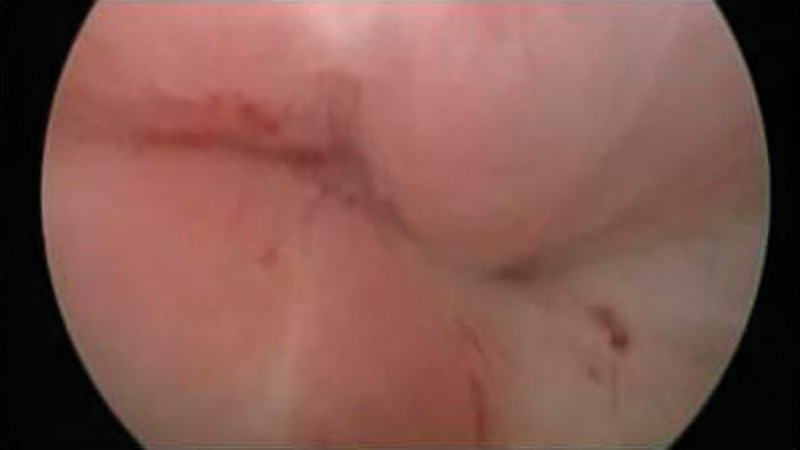
Fistulous orifice viewed through cystoscopy.

On account of the persistence of symptoms, surgical treatment was proposed. She was then sent to another physician (RAMJ) for this treatment. The physical examination in the first visit with this physician showed no abnormalities; there was no urine leakage trough the uterine cervix or the vagina. She already had the diagnosis of adenomyosis, and did not plan to become pregnant again. A surgical correction of the fistula associated with a hysterectomy including the cervix was proposed, which met with the approval of the patient. The decision to perform a hysterectomy was mainly due to a concern about the risk of recurrence, although there is no evidence in the literature that this conduct diminishes this risk. The surgery went as planned. The fistulous tract was excised, and the bladder was sutured in two layers. Fig. 2 depicts the surgical specimens with the uterus and the bladder wall showing the fistulous orifices (Fig. 2). There were no adverse events. The patient was discharged in the third postoperative day. She remained with a bladder catheter for 14 days. After the removal of the catheter, she had no more symptoms. By the time of this publication, the patient will have been asymptomatic for two years after surgery.

### Search Procedure

We have accessed the MEDLINE, BIREME and LILACS databases, searching for articles containing the terms *syndrome of Youssef*; *Youssef syndrome*; *Youssef's syndrome*; *vesicouterine fistula*; *vesico-uterine fistula*; *vesico uterine fistula*; *uterovesical fistula*; *utero-vesical fistula*; and *utero vesical fistula*. In the BIREME and LILACS databases, we have also searched for articles containing the following terms in Portuguese: *síndrome de Youssef*; *fístula vesicouterina*; *fístula vesico-uterina*; *fístula vesico uterina*; *fístula uterovesical*; *fístula útero-vesical*; *fístula útero vesical*. We have also accessed the references of the articles found through the search. The review was conducted between March 2017 and February 2018. The articles that are discussed in the present text were chosen considering their relevance, at the discretion of the authors.

### General Aspects

Vesicouterine fistulae are uncommon complications. Hodonou et al^5^ reported 17 cases in 7 years in a urologic center in Cotonou, Benin. Jóźwik and Jóźwik,^3^ in a review published in 1997, found 602 cases reported in the literature from the 19th century to December 1995; they conducted an additional review (which has not been published), and found 194 additional cases up to December 1997, totalizing 796 cases.^3^ The frequency of vesicouterine fistulae in relation to other urogenital fistulae, as previously reported, varies from 1% to 16.4%.^1^ ^6^ It is very likely that there is an increase in the relative frequency of this type of fistula, because of the increase in cesarean section rates.

In 1957, Youssef^4^ reported a case of amenorrhea and cyclic menouria secondary to a vesicouterine fistula, in the absence of urinary incontinence. In 2000, Jóźwik and Jóźwik^7^ proposed a classification of vesicouterine fistulae, based on the clinical presentation, in three types. Type I presents with amenorrhea and cyclic menouria without urinary incontinence; type II presents with cyclic menouria, but has regular menses and urinary incontinence; and type III, which presents with only urinary incontinence, without menouria and with normal menses. In both types II and III, the urinary incontinence is caused by the passage of urine from the bladder to the uterine cavity, thence trough the cervical canal and the vagina to the vulva. The Youssef syndrome would then correspond to the type I of this classification. Some authors state that the expression cyclic menouria is more adequate than cyclic hematuria, because the material eliminated with the urine is not exactly blood, but rather, menstrual blood, endometrial residua and blood clots.^7^ The present case is a perfect illustration of this concept: the patient presented two episodes of urethral obstruction with blood clots and menstrual particles. Some women with this kind of fistula have recurrent urinary tract infection.^7^ ^8^ ^9^ The urinary incontinence may be intermittent.^7^ ^10^ There are reports of bladder endometriosis associated with this condition, and endometriosis is part of the differential diagnosis.^6^ ^7^ ^11^ The elimination of lochia, along with urine, during the puerperium, which also occurred in the present case, is termed “lochiauria.”^7^ ^12^ Youssef proposed a sphincter/valve mechanism, exerted by uterine isthmus, to explain the absence of urinary incontinence in part of the women with this condition.^4^ Recently, another hypothesis, not necessarily excluding the previous one, was proposed: studies of menstrual physiology show that, during most part of the menstrual cycle, the intrauterine pressure is greater than the intravesical pressure. Only during a small fraction of the cycle, the intravesical pressure becomes greater than the intrauterine pressure when the woman is voiding. This mechanism could explain the intermittent incontinence related by some women.^13^ In some publications, cases described as Youssef syndrome do not correspond exactly to the definition proposed by this author, because they present, besides menouria, also urinary incontinence.^14^ ^15^ ^16^ However, this does not make the reports less interesting, because the causes and the steps for diagnosis and treatment are very similar, with or without urinary incontinence. The present report, if we do not consider the few episodes of scant vaginal bleeding the patient presented, can be considered a case of Youssef syndrome.

### Etiology

Currently, most of the vesicouterine fistulae are secondary to cesarean sections. In a review including reports from 1986 to 1997, 83% of those fistulae were caused by this intervention.^6^ Some mechanisms are proposed to explain how cesarean sections could cause this kind of fistula: 1) non-detected bladder rupture during emergency cesarean, usually with the fetal presentation already engaged, caused by insufficient dissection and/or insufficient drainage of the organ; 2) inadvertent application of a suture in the basis of the bladder, during the suture of the uterus, which can also be associated with insufficient dissection/mobilization of the bladder; 3) abnormal blood supply to the base of the bladder, secondary to an abnormal vascular bed due to multiple dissections, usually after repeat cesareans.^1^ ^6^ ^14^ ^17^ ^18^ In the more recent series, most of the cases occur after repeat cesareans, such as the case in the present report.^7^ ^18^ ^19^ Jóźwik and Jóźwik^7^ reported a significant increase in the proportion of vesicouterine fistulae caused by repeat cesareans in two different periods: 29.6% (21/71) from 1950 to 1985 versus 58.3% (14/24) from 1983 to 1994 (*p* = 0.013).^1^ Many other etiologies are described: prolonged labor; use of forceps; normal birth after previous cesarean; normal birth without previous cesarean; operative vaginal birth with vacuum extractor; placenta percreta; manual extraction of the placenta after normal delivery in a woman with previous cesarean; basiotripsy; excision of Gartner cyst; anterior colporrhaphy; endometrial ablation; excision of uterine fibroma after spontaneous necrosis of uterine fibroma; radiotherapy; pelvic trauma; arterial embolization for the treatment of uterine fibroma (in one of the cases, the symptoms presented one year after the procedure); migration of an intrauterine dispositive; invasion of a malignant tumor; tuberculosis; and actinomycosis.^1^ ^5^ ^6^ ^8^ ^18^ ^20^ ^21^ ^22^ ^23^ ^24^ ^25^ ^26^ ^27^ ^28^ ^29^ ^30^ There is one report after cervical cerclage with Shriodkar procedure and another with modified McDonald procedure. Some cases of vesicouterine fistulae are secondary to congenital malformations of the genitourinary tract.^31^ ^32^ ^33^

### Fertility

There are few studies on fertility and the prognosis of pregnancy in women with vesicouterine fistulae. Lotocki et al^34^ studied 16 women in reproductive age who had their fistulae corrected. Four of these women planned another pregnancy, and all four achieved a full-term successful pregnancy, one of them twice. There was one case of unwanted pregnancy, which was interrupted. Many women in this series did not want another pregnancy, fearing a recurrence of the fistula. Rao et al^8^ reported 3 successful pregnancies that reached full-term in 8 out of 12 women of their series who had their vesicouterine fistulae corrected and were not submitted to tubal sterilization. The report does not inform, however, how many of these eight tried to get pregnant. All three had cesarean deliveries, the authors argue that this was done to avoid recurrence of the fistula. Ali-El-Dein et al^14^ reported 5 pregnancies with good results in a series of 22 women submitted to surgical correction, but here again, they did not inform how many of these 22 tried to get pregnant. Other authors described some isolated cases of successful pregnancies after the treatment of vesicouterine fistulae, usually after surgical correction. These reports suggest that fertility can usually be recovered after treatment of this kind of fistula. In cases of pregnant women who underwent vesicouterine fistula correction, Yip et al^6^ recommend delivery through cesarean to lower the risk of recurrence (of the fistula), but they do not present arguments or references to support this recommendation. We did not find reports about pregnancy in women with non-corrected vesicouterine fistulae. However, we also did not find studies that accessed specifically this question. We may suppose that fertility is impaired by this condition, especially in cases with urinary incontinence, because this symptom usually implies the passage of urine through the uterine cavity. There is a curious report about the deliberate creation of a vesicouterine fistula as a reversible contraceptive method in Sweden.^35^

### Diagnosis

The first step in the diagnosis is, of course, a suggestive clinical presentation. The absence of urinary incontinence can make the diagnosis more difficult. There are some reports of delayed presentation of the symptoms. Ugurlucan et al^35^ report a case of a 55-year-old woman who presented with urinary incontinence 30 years after a cesarean section. They state that a vesicouterine fistula should be part of the differential diagnosis of urinary incontinence. There are some reports of unusual clinical presentations: Kamil and El Mekresh^36^ report a case of protrusion of the umbilical cord through the urethral meatus. The patient, a 31-year-old woman, had a bladder injury during her second cesarean section, which was performed at 24 weeks of gestation for antepartum hemorrhage. The immediate correction was performed by a urologist. She remained without symptoms after these procedures, until 21 weeks of the next pregnancy, when she presented with fetal demise and cord prolapsed through the urethra. She was rapidly submitted to a cesarean and surgical correction of the fistula. The authors made the hypothesis that the fistulous orifice was reopened due to the distention of the uterine wall during pregnancy.^36^ Lesovoy et al^37^ reported a case of “fetus in the bladder.” A 39-year-old woman had a bladder injury during her second cesarean, which was promptly corrected. She was discharged without symptoms, but three months later, she presented with symptoms suggestive of vesicouterine fistula, and the diagnosis was confirmed through a cystoscopy. The attending team offered her surgical treatment, but she refused. In her next pregnancy, she was admitted to a general hospital with lower abdominal pain and urinary symptoms. The ultrasound scan showed a missed abortion with 11 weeks of gestation inside the bladder plus a vesicouterine fistula. After a cystoscopy, which confirmed a 2-cm fistula, she was submitted to surgical correction, with a good outcome.^37^ Keskin et al^38^ reported a case of a woman who presented cyclic menouria for 20 years after her 2nd cesarean before the diagnosis was made.^38^ The present report is a good example of the difficulty in diagnosis, because it was made about two years after the beginning of the symptoms; we have to emphasize that, during this period, the woman looked for medical care four times, consulting with different physicians, before a diagnosis was determined. The confirmation of the diagnosis can be made through cystoscopy (sometimes after the injection of dye in the bladder), cystography, intravenous pyelography, hysterography, sonography, and other types of imaging exams.^6^ ^8^ ^10^ Very often, only one of these exams is not sufficient for the diagnosis; in some occasions, two or more are necessary.^39^ ^40^ Additionally, it is recommendable that, besides confirming the diagnosis, the attending physician try to define precisely the size and course of the fistula, and also try to exclude the presence of other concomitant fistulae. Because of this, it is usual to perform two or more exams during the evaluation. Porcaro et al^10^ recommend routine intravenous pyelography to allow a view of the entire urinary tract. There are recent reports about diagnosis with computed tomography and nuclear magnetic resonance; those methods, however, are not usually mandatory.^14^ ^41^ ^42^

### Treatment

The proposed treatments for vesicouterine fistulae are: 1) surgical resection - a) through abdominal open surgery. This is the most common surgical treatment for this disease. If there is indication for hysterectomy, it must be performed in the same surgical procedure. However, hysterectomy is not necessary if the only intention is the correction of the fistula.^10^ If hysterectomy is not performed, the placing of an omentum layer between the uterus and the bladder to diminish the risk of recurrence of the fistula is recommended.^10^ In the experience of Kottász and Gergely,^43^ the surgeries performed by mixed teams, composed by gynecologists and urologists, have the best results; b) laparoscopic correction; Maioli et al^44^ state that the final result of the laparoscopic treatment is very dependent on the experience of the surgical team with this technique; c) there are reports of good results with correction through the vaginal route; d) there are recent reports of good results with robotic surgery.^43^ ^44^ ^45^ ^46^ ^47^ ^48^ ^49^ In the surgical treatment, the choice of the route, namely open abdominal, laparoscopic, transvaginal or robotic, depends on the location of the fistula and, as stated before, on the experience of the team in non-conventional routes (laparoscopic, transvaginal or robotic). The best results reported are with the open abdominal route.^10^ 2) Molina et al^50^ report a good result with fulguration of the fistulous tract through cystoscopy in a small fistula. Tarhan et al^9^ also report a good result with this treatment but associated with bladder drainage and hormonal suppression of menstruation. 3) There are many reports of conservative treatment and spontaneous cure. Jóźwik and Jóźwik,^3^ in their review, found 29 cases of spontaneous resolution (excluding 19th century reports), including a case of their own. The proportion of spontaneous cure was of 5.1% of all the cases they found in the literature (41/796). However, in the 9 cases in their review that were submitted to hormonal suppression of menstruation with progestogens or combined oral contraceptives, 8 had their fistulas cured (88.9%). In the remaining 787 women without hormonal suppression, 33 were cured (4.2%). They compared those proportions (88.9% versus 4.2%) with the Chi-squared test and concluded that the difference was significant (*p* < 0.001). There is one report of menstrual suppression with a gonadotrophin-releasing hormone analogue.^51^ If one tries the conservative approach associated with hormonal suppression of menstruation, the results are better if the suppression is initiated soon after the diagnosis.^8^ The chance of cure with a conservative approach diminishes if there is urinary incontinence.^6^ If incontinence is present and a conservative approach is the option, the patient should remain with continuous bladder drainage for at least three months.^10^ If the surgical correction is not performed in the first few days after the cesarean section, it is recommended to postpone it for two to three months, to allow for uterus involution and complete resolution of the inflammatory process related to scar formation.^10^

### Reports from Brazil

Five articles from Brazil were found in the review; altogether, they report eight cases. Oliveira et al^52^ report a vesicocervical fistula developed after an abdominal hysterectomy with preservation of the cervix. In the article by Carvalho et al,^53^ the three cases are of obstetrical origin: two after cesarean and one after uterine rupture. Agostinho et al^54^ report two cases, one after cesarean and other after vaginal delivery with abnormal course. Lopes et al^55^ report a surgical correction of a congenital vesicouterine fistula. Maioli et al^44^ report a case corrected through laparoscopy. All the eight cases reported were surgically corrected, with good results.

## Strength and Limitation of the Present Study

The limitation of the present study is the fact that the authors conducted the final treatment but were not involved with the care regarding the delivery and the following two years, during which the patient presented symptoms and did not have the correct diagnosis. The information about this period was given by the patient herself, since we did not have access to the records of the several professionals who had previously examined her.

The strength of the study is that it reports a case with a not-so-obvious diagnosis, with the potential to lead professionals to wrong diagnoses and improper interventions. It is very likely that cases similar to this one will become more common, especially in Brazil, due to the high rates of cesarean sections in the country.

## Take-Away Lessons of the Case Report

A vesicouterine fistula is one of the possible diagnoses if urinary symptoms (like hematuria and urinary incontinence) develop after a cesarean section.

A vesicouterine fistula is one of the possible long-term morbidities secondary to cesarean sections.

## Conclusion

Apart from the already known higher mortality and short term morbidity associated with cesarean sections, as well as the long-term risks, such as placenta previa, placental abruption and uterine rupture in future pregnancies, the risk of genitourinary fistula, especially vesicouterine fistula, must be taken into account in the evaluation of the risks associated with abdominal delivery.^56^ ^57^ ^58^ ^59^ ^60^ Taking into account the reports in the literature and also the present case, we can conclude that vesicouterine fistulae have a fairly good prognosis, provided the diagnosis can be correctly made. Almost all patients have had their fistulae cured, most of them with surgical treatment. However, delays in the diagnosis were common.
